# Investigation of the essential role of capsular polysaccharide in serotype 1 *Streptococcus pneumoniae*

**DOI:** 10.3389/fcimb.2026.1835100

**Published:** 2026-06-10

**Authors:** Vanessa S. Terra, Fauzy Nasher, Elisa Ramos-Sevillano, Si Yin Tan, Alexander A. Smith, Lok-To Sham, Jeremy S. Brown, Brendan W. Wren

**Affiliations:** 1 Department of Infection Biology, London School of Hygiene and Tropical Medicine, London, United Kingdom; 2 Centre for Inflammation and Tissue Repair, Department of Medicine, Royal Free and University College Medical School, Rayne Institute, London, United Kingdom; 3 Infectious Diseases Translational Research Programme, Yong Loo Lin School of Medicine, National University of Singapore, Singapore, Singapore; 4 Department of Microbiology and Immunology, Yong Loo Lin School of Medicine, National University of Singapore, Singapore, Singapore

**Keywords:** capsule, mutagenesis, pneumococcal infection, *S. pneumoniae* (*Streptococcus pneumoniae*), serotype 1, zebrafish

## Abstract

*Streptococcus pneumoniae* remains a major global cause of morbidity and mortality, responsible for an estimated half million deaths annually despite widespread implementation of pneumococcal conjugate vaccines. Notably, invasive pneumococcal disease (IPD) caused by serotype 1 persists and disproportionately affects vulnerable populations in low-resource settings, despite inclusion of this serotype in available vaccines. Serotype 1 exhibits atypical biology characterized by short nasopharyngeal carriage and high invasive potential. This phenotype has been linked to capsule-mediated evasion of host immunity, including resistance to phagocytosis, complement deposition, opsonophagocytic killing, mucus entrapment, and neutrophil extracellular traps. The zwitterionic serotype 1 capsular polysaccharide is proposed to enhance traversal of the mucus layer and access to deeper tissues such as olfactory epithelium and brain. The genes required for capsule biosynthesis are located within the *dexB–aliA* cps locus; however, serotype 1 additionally requires genes outside this locus for AATGal biosynthesis and transfer to undecaprenyl phosphate (UndP). To elucidate the role of the serotype 1 capsule in pathogenicity, we performed genetic analysis and mutagenesis of *cps*-linked genes. Genes involved in capsule biosynthesis and assembly appear to be essential for viability because they could not be disrupted despite multiple attempts, unlike other genes on the capsule loci but not involved in capsule biosynthesis such as *rlmA*. To overcome the inability of constructing defined *cps* mutants, we used a serotype 2 capsule switch (D39::cps1) and an *E. coli* strain expressing the *cps1* locus and generated defined capsule mutants (*ΔwchB, ΔwchD, Δgla, Δugd*). We used these mutants to quantify capsule thickness by FITC-dextran labeling and electron microscopy, measured complement deposition and antibody binding, and evaluated virulence in the Zebrafish infection model relative to the parental serotype 1 strains 519/43 and D39WT. Here, we report that although the D39::cps1 strain expresses a serotype 1 capsule and retains key associated properties—including increased capsule thickness and reduced antibody binding and complement deposition—its full virulence is not recapitulated. Instead, the capsule-switched strain exhibited significantly attenuated virulence compared with the parental serotype 1 strains suggesting that virulence is not entirely dependent on the capsule.

## Introduction

**Figure 1:**
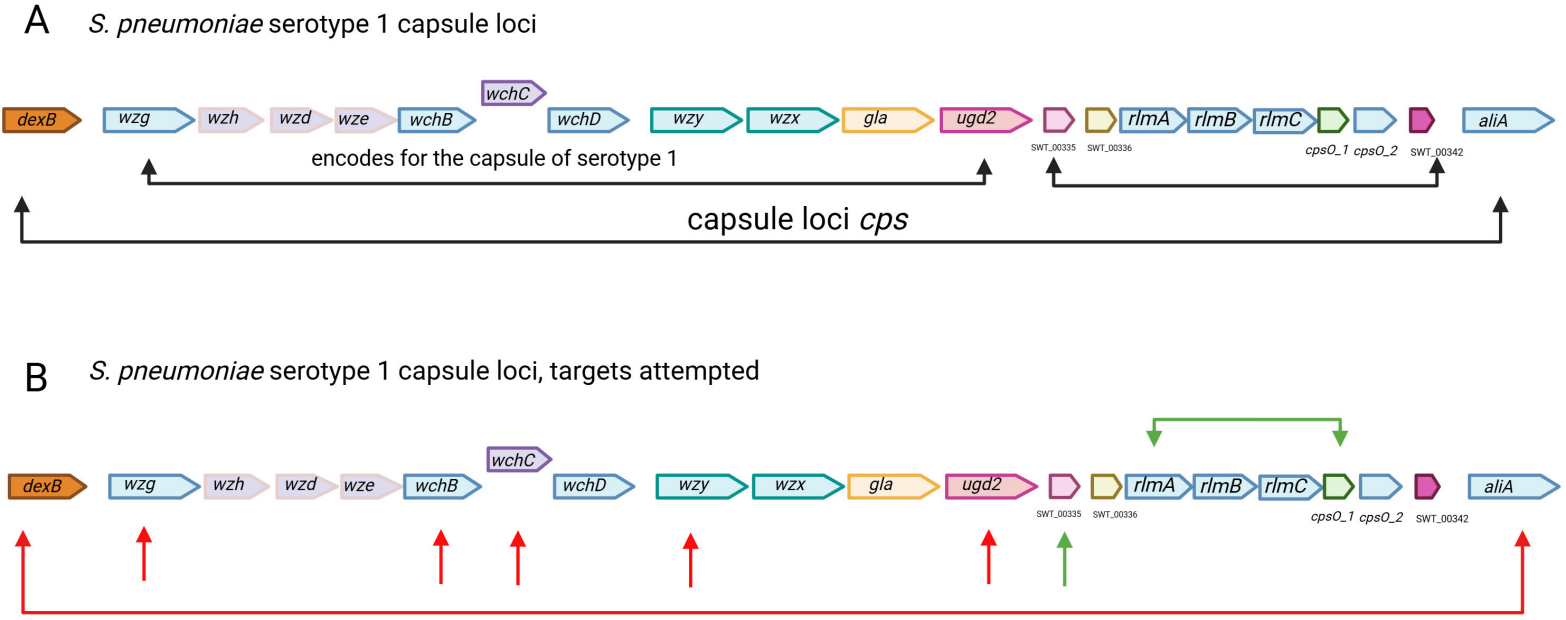
**(A)** S. pneumoniae serotype 1 capsule loci organization. Figure adapted from Bentley et al., 2002. **(B)** Red arrows denote the targets attempted unsuccessfully. Green arrows denote the mutants made in cps1 loci. SWT_00335 codes for an hypothetical protein.

**Figure 2:**
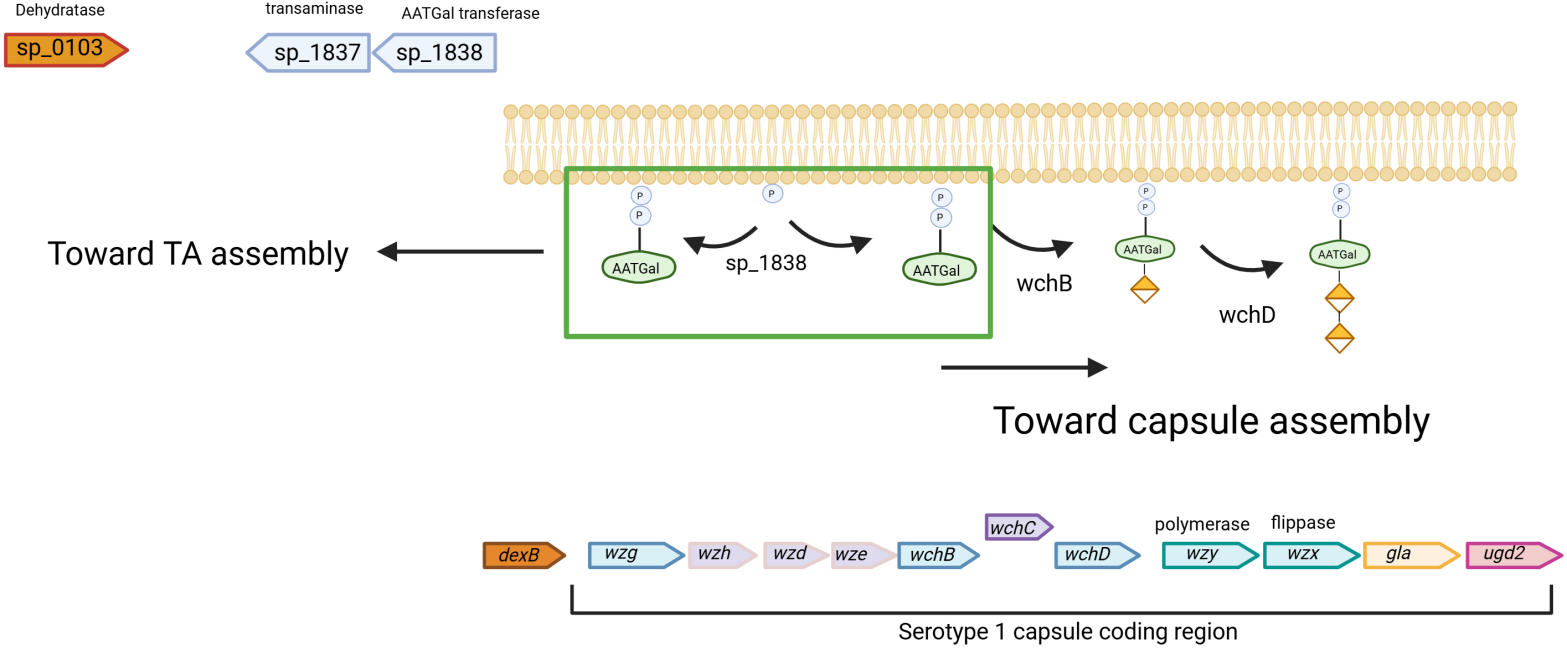
Model depicting AATGal metabolism for teichoic acid (TA) and capsule biosynthesis in Streptococcus pneumoniae serotype 1. The dehydratase sp_0103, transaminase sp_1837, and AATGal transferase sp_1838 are predicted to function sequentially in the synthesis and transfer of 2-acetamido-4-amino-2,4,6-trideoxygalactose (AATGal). Within the membrane (green box), sp_1838 mediates the transfer of AATGal onto a lipid-linked intermediate. This intermediate can be directed toward TA assembly (left), contributing to cell wall teichoic acid structures, or alternatively toward capsule biosynthesis (right), where AATGal-modified precursors are incorporated into the growing capsular polysaccharide. The lower panel depicts the serotype 1 capsule biosynthetic locus, including regulatory and glycosyltransferase genes (wzg–wze, wchA, wchB, wchC, wchD), polymerase (wzy), flippase (wzx), and galacturonic acid-producing enzymes (gla, ugd2), highlighting the genetic context for capsule production.

*Streptococcus pneumoniae* (pneumococcus) is usually a human commensal colonizing asymptomatically the nasopharynx, but it is also an opportunistic pathogen able to cause pneumonia, meningitis, and bacteremia making it a major global burden. Despite the widespread use of highly effective conjugate vaccines, *S. pneumoniae* remains responsible for more than one million deaths annually, including approximately 300,000 among children under 5 years of age (WHO, September 9, 2024). Of note is the persistence of invasive pneumococcal disease rate caused by *S. pneumoniae* serotype 1 (Kwambana-Adams et al., 2016; Bar-Zeev et al., 2021) (a serotype included in most available vaccine formulations) particularly in low-resource settings and in the vulnerable population (Johnson et al., 2010). To date, more than 100 pneumococcal serotypes have been identified (Bentley et al., 2006; Ganaie et al., 2020), each exhibiting varying capacities to colonize the nasopharynx (Abdullahi et al., 2012) or to cause invasive pneumococcal disease (Brueggemann et al., 2003). Unlike other serotypes, serotype 1 strains are inefficient asymptomatic colonizers of the nasopharynx and instead present with a relatively high invasive potential (invasiveness) (Balsells et al., 2018); thus, serotype 1 strains do not seem to behave like a typical commensal *S. pneumoniae* strain (Weiser, 2010; Ritchie et al., 2012) starting by being able to establish a very short colonization lasting only 1 to 2 weeks (Abdullahi et al., 2012; Dube et al., 2018; Chaguza et al., 2021). Other studies have shown a positive correlation between degree of encapsulation, carriage duration, and invasive potential (Chaguza et al., 2016). The pneumococcal capsule, regarded as one of the most critical virulence factors for pathogenicity (Hyams et al., 2010a), has been shown to confer protection against multiple host defense mechanisms, including phagocytosis (Kadioglu and Andrew, 2004), complement mediated immunity (Hyams et al., 2010b), and opsonophagocytic killing (Melin et al., 2010b). It also reduces capture by neutrophil extracellular traps (Wartha et al., 2007) and limits entrapment within airway mucus, thereby reducing mucus-mediated clearance (Nelson et al., 2007). When comparing the serotype 1 capsular polysaccharide with all other known *S. pneumoniae* serotypes, this is the only reported zwitterionic capsule which can potentially facilitate transversing the mucus layer to reach the underlying epithelium (Leal et al., 2017). The serotype 1 capsule was previously found to be significantly more resistant to opsonization and complement deposition (Melin et al., 2010a). An enhanced capability of transversing the nasopharynx and reaching the olfactory tissues and eventually reaching the brain compartment was observed (Jacques et al., 2020). Serotype 1 strains express a capsule that is different from all other known serotypes; therefore, a more detailed fundamental analysis is needed to understand how these differences contribute to its enhanced virulence and tissue tropism. In *S. pneumoniae* serotype 1, the genes required for capsule assembly are organized on the chromosome within a genomic region bounded by *dexB* and *aliA*, a conserved arrangement shared by many pneumococcal serotypes (Jiang et al., 2001). The locus is known as the *cps locus* (Muñoz et al., 1997). The genes within the *cps* loci are organized based on their functions (Yother, 2011; Geno et al., 2015). The first group, encompassing four genes common to all serotypes, comprises the modulatory genes (*wzg*, *wzh*, *wzd*, and *wze* also known as *cpsA cpsB cpsC* and *cpsD*) (Guidolin et al., 1994). Immediately downstream are the serotype-specific genes encoding for the glycosyl transferases (*wchB* and *wchD*) and the acetylase (*wchC*) (Muñoz et al., 1997) (**Figure 1**). However, in contrast to most serotypes, serotype 1 relies on three genes located outside the capsule locus for the generation and transfer of the first sugar, AATGal, to the lipid carrier (UndP); these genes are instead found elsewhere in the genome and are shared with the teichoic acid (Fischer et al., 1993) and lipoteichoic acid (Behr et al., 1992) biosynthesis pathway. These genes—sp_0103, sp_1837, and sp_1838—encode enzymes involved in the biosynthesis and transfer of UDP-AATGal. The pathway begins with sp_0103, which encodes a UDP-N-acetylglucosamine 4,6-dehydratase. This enzyme catalyzes the conversion of UDP-GlcNAc to UDP-4-keto-6-deoxy-D-GlcNAc, a key intermediate in the pathway. Next, sp_1837, encoding a UDP-4-amino-4,6-dideoxy-N-acetyl-β-L-altrosamine transaminase, transfers an amino group to the C-4 position of this intermediate, producing UDP-AATGal. The product is transferred to the lipid carrier undecaprenyl phosphate by the AATGal transferase encoded by sp_1838, enabling incorporation into downstream capsule or teichoic acids biosynthesis (Aanensen et al., 2007) (**Figure 2**).

Additionally, serotype 1 carries both *ugd* and *Gla* which are responsible for the synthesis of UDP-Glc*p*A (glucuronic acid) which is then converted into UDP-GalpA (galacturonic acid) respectively (Muñoz et al., 1997; Jiang et al., 2001; Bentley et al., 2006). Serotype 1 also contains rhamnose synthesis genes; however, there is a frameshift in *rlmD* which results in a type 1 capsule devoid of rhamnose (Muñoz et al., 1997).

**Figure 3:**
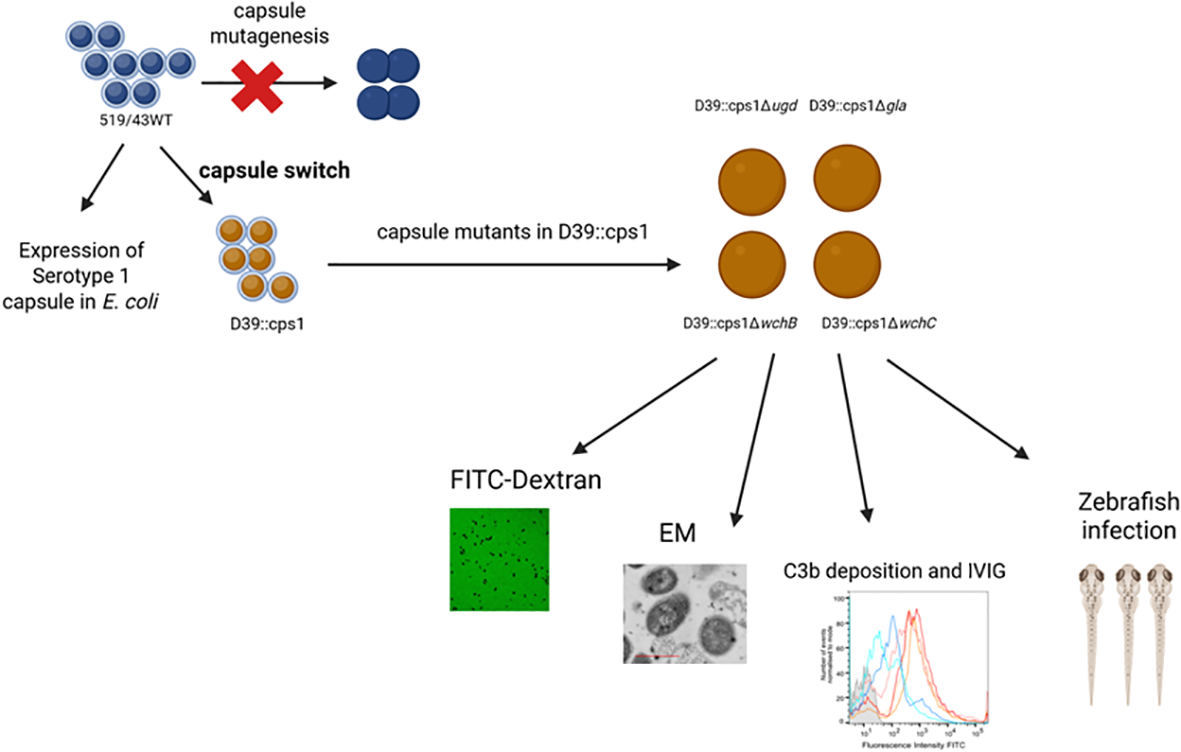
Graphic diagram depicting the experimental flow of the project.

To elucidate the role of the capsule in pathogenesis and to define the functions of the enzymes involved, we conducted a comprehensive analysis of the *dexB–aliA* genomic region. Several genes in this pathway could not be disrupted after repeated attempts, and therefore, they are likely to be essential for viability. In contrast, other genes in this region were successfully mutated in the serotype 1 strain 519/43. To address this limitation, two strains were generated: an *E. coli* strain carrying the *S. pneumoniae* serotype 1 capsule loci able to generate a full capsule, and a D39W strain harboring the *cps1* locus (D39::cps1). Targeted mutations in *wchB*, *wchD*, *gla*, and *ugd* produced defined capsule-deficient variants in both strains. The resulting strains—D39WT, D39::cps1, and D39::cps1Δ*wchB*—were subsequently used to assess the contribution of the serotype 1 capsule to virulence, complement deposition, and antibody binding. Capsule thickness in D39::cps1 was quantified and compared with that of strain 519/43 using FITC-dextran labeling and electron microscopy. Finally, the role of the serotype 1 capsule in virulence was evaluated by monitoring zebrafish larval survival following infection with D39::cps1, D39::cps1*ΔwchB*, and the parental serotype 1 strain 519/43 (**Figure 3**).

## Methods

*Streptococcus pneumoniae* serotype 1 strain 519/43 **Table 1** ST5316, isolated in 1943 in Denmark, acquired from the Statens Serum Institute, and serotype 1 strain BVJ1JL ST5012 isolated from a Malawian patient (Swarthout et al., 2020) were used in this study. Routinely, pneumococci were grown in brain heart infusion (BHI, Oxoid) broth, on blood agar plates (BAB, Oxoid) supplemented with 5% (v/v) defibrinated horse blood or on tryptic soy agar-II plates supplemented with 5% (v/v) sheep blood (blood agar; Biomed Diagnostics, 221261) under microaerophilic conditions at 37°C. Where appropriate, 100 µg/mL spectinomycin, 250 µg/mL for kanamycin, 150 µg/mL for spectinomycin, and 300 µg/mL for streptomycin were added to the culture media.

**Table 1:** Strains used and generated in this study.

Strains	Characteristics	Origin
D39 WT (serotype 2)		
D39::cps1	CPS1 *rpsL1* CEP::P-*kan*-*rpsL*^+^-barcode	(Chun et al., 2023)
D39::cps1*ΔwchB*	CPS1 *rpsL1* CEP::P-*kan*-*rpsL*^+^-barcode *ΔwchB*	This study
D39::cps1*ΔwchC*	CPS1 *rpsL1* CEP::P-*kan*-*rpsL*^+^-barcode *ΔwchC*	This study
D39::cps1*Δgla*	CPS1 *rpsL1* CEP::P-*kan*-*rpsL*^+^-barcode *Δgla*	This study
D39::cps1*Δugd*	CPS1 *rpsL1* CEP::P-*kan*-*rpsL*^+^-barcode *Δugd*	This study
519/43 (serotype 1)	ST5316	(Lund, 1951)
BVJ1JL (serotype 1)	ST5012	(Swarthout et al., 2020)
*E. coli Falcon*	W3110 Δ*lpxM*, *ΔwecA-wzzE*(*gne*)	(Kay et al., 2022)

### Generation of constructs for mutagenesis of strain 519/43

Constructs targeting genes within the polysaccharide biosynthetic region essential for capsule assembly (*wzg*, *wchB*, *wchC*, *wzy*, and *ugd_2*), as well as genes located outside the essential region but within the *dexB–aliA* locus (*dexB* and *aliA, rlmA*, *rlmB*, *rlmC*, *cpsO_1*, and *swt_00335*), were generated following the protocol described by Terra et al. (2020), with minor modifications (Terra et al., 2020b, 2020a). Mutagenesis constructs were assembled using Gibson Assembly (New England Biolabs, UK). Briefly, the plasmid backbone, spectinomycin resistance cassette, left homology arm (LHA), and right homology arm (RHA) were amplified with overlapping homologous ends for each construct using the primers listed in **Tables 2** and **3**. The resulting amplicons were combined in a Gibson Assembly reaction containing 10 µL of master mix, 2 µL of each DNA fragment (backbone, spectinomycin cassette, LHA, and RHA), and 2 µL of nuclease-free water and incubated at 50°C for 1 h. Each construct was subsequently transformed into chemically competent *E. coli* C2987 (New England Biolabs, UK). Colonies resistant to both ampicillin (plasmid backbone) and spectinomycin were selected and grown overnight for plasmid DNA extraction. The resulting plasmids were designated pVT_*wzg*_spec, pVT_*wchB*_spec pVT_*wchC*_spec, pVT_*wzy*_spec pVT_*ugd_2*_spec, pVT_*rlmA_O1*_spec, and pVT_*swt_00335*_spec.

**Table 2:** List of primers generated in this study to mutate the capsular polysaccharide coding region, particularly the region that encodes for the capsule synthesis and assembly as well as two targets outside of the region responsible for the synthesis and assembly.

Primer name	Sequence 5’-3’	Origin
pGEM_wzg FW	gggtagataaccctatagtgagtcgtattac	This study
pGEM_wzg_rev	ttaaaacgtctcatggtcatagctgtttc	This study
LHA wzg_fwd	atgaccatgagacgttttaaaaaatcacgttc	This study
LHA wzg_rev	cgggggatccgatatcagctagtagtttttgaatattttc	This study
spec_wzg_fwd	agctgatatcggatcccccgtttgatttttaatg	This study
spec_wzg_rev	atcgcacataggatccaatttttttataatttttttaatctgttatttaaatag	This study
RHA wzg_fwd	aattggatcctatgtgcgattgaacttc	This study
RHA wzg_rev	cactatagggttatctaccctccatcac	This study
pgemT_dexB_aliAfwd	tctcaactggccctatagtgagtcgtattac	This study
pgemT_rev_dexB_aliA	agcaaattcatagtgtcacctaaatagcttg	This study
aliA_fwd	ggtgacactatgaatttgctaaagctaaatc	This study
aliA_rev	cgggggatccttatttcacatgttttgcg	This study
spec_cassette_fwd	tgtgaaataaggatcccccgtttgatttttaatg	This study
spec_cassette_rev	tttcttgcatggatccaatttttttataatttttttaatctgttatttaaatag	This study
dexB_fwd	aattggatccatgcaagaaaaatggtggc	This study
dexB_rev	cactatagggccagttgagatccggctg	This study
pGEMT_wchB_fwd	agataaaataccctatagtgagtcgtattac	This study
pGEMT_wchB_rev	gtaaattcattagtgtcacctaaatagcttg	This study
wchB LHA_fwd	ggtgacactaatgaatttacaaaagaaaaaaattatgttaataac	This study
wchB LHA_rev	atccaaacatgaaattctaaacagtaataacacc	This study
Spec_wchB_fwd	ttagaatttcatgtttggatcaggagttg	This study
Spec_wchB_rev	cttttaatatccttataatttttttaatctgttatttaaatagtttatag	This study
wchB RHA FW	aaaattataaggatattaaaagaaaaaggtatagatac	This study
wchB RHA rev	cactatagggtattttatctaaataagcatctataataatttg	This study
pGEM_rlmnREV	acctttcatatagtgtcacctaaatagcttg	This study
rlmA_fwd	ggtgacactatatgaaaggtattattctagcaggtg	This study
rlmA_rev	cgggggatccacagtcgcacctttctctttc	This study
spec_fwd	gtgcgactgtggatcccccgtttgatttttaatg	This study
spec_rev_rlmn	ttaaaatcatggatccaatttttttataatttttttaatctgttatttaaatag	This study
CPS_01_fwd	aattggatccatgattttaattacaggggc	This study
CPS_01_rev	ccagttgagactagtccgtagaaatataaactag	This study
pGEM_rlmn_FW	tacggactagtctcaactggccctatag	This study
pGEM_ugd_fwd	ttgtatatttccctatagtgagtcgtattac	This study
pGEM_ugd_rev	ctattttcatcatggtcatagctgtttc	This study
LHA ugd2_fwd	tatgaccatgatgaaaatagcagtagcag	This study
LHA ugd2_rev	cgggggatcccttttaattattataattgcatcagg	This study
spec ugd2_fwd	taattaaaagggatcccccgtttgatttttaatg	This study
spec ugd2_rev	agatccagcaggatccaatttttttataatttttttaatctgttatttaaatag	This study
RHA ugd2_fwd	aattggatcctgctggatcttatgtagg	This study
RHA ugd2_rev	cactatagggaaatatacaaattgatttaatctctcttaaaaatatc	This study
PGEM_335_fwd	taagtgaattccctatagtgagtcgtattac	This study
PGEM_335_rev	tacaaattgacatggtcatagctgtttc	This study
335LHA_fwd	tatgaccatgtcaatttgtatatttctagtccc	This study
335LHA_rev	cgggggatcctatcagcacctcacttcg	This study
335_spec_fwd	ggtgctgataggatcccccgtttgatttttaatg	This study
335_spec_rev	agaatacgaaggatccaatttttttataatttttttaatctgttatttaaatag	This study
RHA335_fwd	aattggatccttcgtattctgttaatgaattg	This study
RHA335_rev	cactatagggaattcacttacttcatattacatttag	This study

### Transformation of 519/43 with pVT_*gene*_spec

Strain 519/43 was cultured overnight under static conditions at 37°C with 5% CO₂. The following morning, overnight cultures were diluted 1:50 and 1:200. When the optical density at 600 nm (OD_600_) reached 0.06, cells were transformed with 500 ng of pVT_gene_spec following the previously described protocol (Terra et al., 2020b). Transformants were selected on blood agar base plates supplemented with 100 µg/mL spectinomycin and then re-streaked onto fresh spectinomycin-containing plates and incubated overnight at 37°C with 5% CO^2^. Colonies that exhibited growth were inoculated into BHI broth containing 100 µg/mL spectinomycin and grown statically overnight. Chromosomal integration of the mutation was verified by PCR using confirmation primers (**Tables 2**, **3**) annealing upstream and downstream of the targeted region. The mutation was further confirmed by sequencing the corresponding amplicon with the primers listed in **Tables 2** and **3**.

**Table 3:** Primers used for complete deletion of gene *wchC*, done exactly as described in Terra et al., 2020b.

Primer name	Sequence 5’-3’	Origin
wchBfW	TTT GCGGCCGC GGCTGCTGCCCAAATTATTAAGA	This study
wchBfW	TACTTTCATAATTTCATCTCCCTTGATGGATTC ATCCTATTCATCTAAAGCTCC	This study
wchDfW	GGAGCTTTAGATGAATAGGAT GGATCCATCAAGGGAGATGAAATTATGAAAGTA	This study
wchDRev	TTT GCGGCCGC TGGGATTCTTACTGGTATCATAGTTTAAAGG	This study

### Disruption of *Streptococcus pneumoniae* serotype 1 capsule biosynthesis in *Escherichia coli*

*Streptococcus pneumoniae* serotype 1 capsule biosynthesis locus was expressed in *Escherichia coli* strain Falcon (Kay et al., 2022) harboring the required plasmids (pBeloBAC11 and pEC415_AATGal) using chloramphenicol (15 µg/mL) (constructed elsewhere by Dr Emily J. Kay personal communication, manuscript in preparation). To modify the capsule locus within pBeloBAC11, targeted deletion and reassembly were performed using Gibson assembly. Primers (**Table 4**) were designed to amplify the plasmid backbone while excluding the region of interest, incorporating homology arms corresponding to the *wchB* and *ugd* genes of the *S. pneumoniae* capsule locus to facilitate seamless reassembly. Amplified fragments were assembled using the NEB HiFi DNA Assembly Master Mix following the supplier’s protocol, generating a modified pBeloBAC11 plasmid. The modified pBeloBAC11 was transformed into chemically competent *E. coli* Falcon cells already containing the required pEC415_AATGal for expression of *S. pneumoniae* serotype 1 capsule (manuscript in preparation). Transformants were selected on chloramphenicol-supplemented (15 µg/mL) and ampicillin-supplemented (100 µg/mL) LB media plates.

**Table 4:** List of primers generated in this study to mutate the *S. pneumoniae* serotype 1 capsule polysaccharide coding region expressed in the *E. coli* strain Falcon.

Primer name	Sequence 5’-3’	Origin
wchBf	GATGAATAGGATTATAAAAAATATTGTTG	This study
wchBr	ATCAAAATAATCTTCCTAAAATATAATTAAC;	This study
ugf	AGAATACTCAAGCTTGCATGCTGTCACAATTTGGAAAC;	This study
ugdr	AGAGTCGACCTGCAGGCATGCTCTCGTTATACATTATAGTAGTC.	This study

### Mutation of genes *wchB*, *wchC*, *ugd*, and *gla* in the D39 capsule switch strain

**Figure 4:**
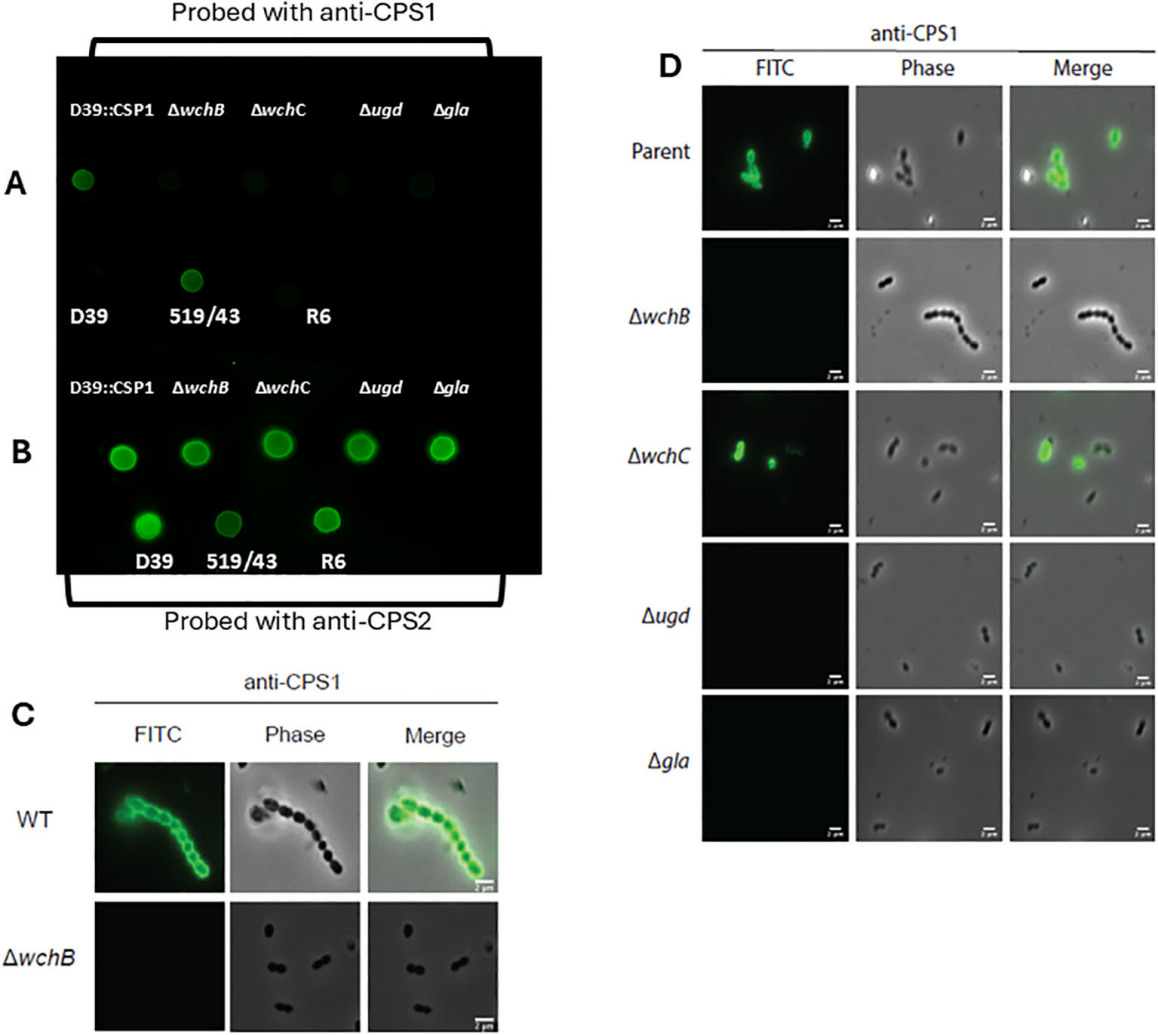
Inactivation of *wchB*, *ugd*, and *gla* abolished capsule production. **(A, B)** Dot blots of serotype 1 isogenic capsule-switch mutant and capsule loci mutants probed with anti-serotype 1 sera and anti-serotype 2 sera, respectively. **(A)** D39::cps1, D39::cps1Δ*wchB*, D39::cps1Δ*wchC*, D39::cps1Δ*ugd*, and D39::cps1Δ*gla* were stained with anti-serotype1 sera (Statens, Denmark), followed by Alexa Fluor anti-rabbit secondary antibodies. **(B)** D39::cps1 and mutants were stained with anti-serotype2 antisera, followed by Alexa Fluor anti-rabbit secondary antibodies. Anti-serotype 2 has wide reactivity and recognizes surface-bound proteins, and therefore, all probed strains and corresponding mutants reacted with it including the negative acapsular R6 strain. **(C, D)** Strains D39::cps1,D39::cps1Δ*wchB*, D39::cps1Δ*wchC*, D39::cps1Δ*ugd*, and D39::cps1Δ*gla* were stained with anti-serotype 1 antisera, and the capsule was detected by labeling with Alexa Fluor 488 conjugated anti-rabbit secondary antibodies. Bar, 2 µm. Δ*wchC* showed partial labeling with anti-CPS1 (54.9%, N = 253) Representative images from three biological replicates are shown. Strains D39::cps1Δ*wchB*, D39::cps1Δ*ugd*, and D39::cps1Δ*gla* showed no reactivity with the anti-serotype 1 antibody confirming the loss of capsule.

The capsule switch strain used for the mutagenesis was constructed as described previously by Chun Ye-Yu et al (Chun et al., 2023). The same methodology was used to construct mutant strains Δ*wchB*, Δ*wchC*, Δ*ugd*, and Δ*gla*. Briefly, amplicons for transforming strain NUS0758 (D39 WT) [CPS1 *rpsL1* CEP::P-*kan*-*rpsL*^+^-barcode] were prepared by PCR using the primers listed in **Table 5**. The purified PCR fragments were assembled by Gibson’s assembly (Gibson et al., 2009) to generate the deletion cassettes. Next, strain D39WT was grown in BHI until the culture reached OD_600_ of 0.2. The culture was diluted to an OD_600_ of 0.03 in 1 mL of BHI. Natural competence was induced by adding 5 µL of 50 ng/mL competence-stimulating peptide 1 (CSP-1), 4 µL of 10% (w/v) bovine serum albumin (BSA), and 1 µL of 1M CaCl_2_. After incubating for 9 min at 37 °C, 9 µL of the assembled cassettes was added and the mixtures were incubated at 37 °C in 5% CO_2_ for 1.5 h. Transformants were suspended in 0.7% (w/v) Difco nutrient agar (Biomed Diagnostics, 213000) and poured on blood agar plates supplemented with the appropriate antibiotics. The surviving colonies were streaked twice on blood agar with the corresponding antibiotics, and the antibiotics cassettes were validated by diagnostic PCR using PowerPol 2X PCR Mix with Dye (ABclonal, RK20719) and Sanger sequencing (**Figure 4**).

**Table 5:** Primers used to construct mutants in *wchB*, *wchC*, *gla* and *ugd* in the capsular switch strain D39::cps1.

Strain	Primer	Sequence
Δ*wchB*	wchB Up F	Gcaagccgtatcgctaact
	wchB Up R	CATTATCCATTAAAAATCAAACGGATCCTAtaacataatttttttcttttgtaaattc
	wchB Dn F	CAAAAGCATAAGGAAAGGGGCCCgataaaataaaaggagctttagatgaatag
	wchB Dn R	Ttgatcaccatcacctgctaaa
Δ*wchC*	wchC Up F	Gagtatgggtatgaagtgctagtg
	wchC Up R	CATTATCCATTAAAAATCAAACGGATCCTAaccaacaatattttttataatcctattc
	wchC Dn F	CAAAAGCATAAGGAAAGGGGCCCaaacgtttatcaagggagatgaaattatgaaag
	wchC Dn R	Caactctggaattcctgctttattag
Δugd	ugd Up F	Tggaattagaaggaacgatgacaa
	ugd Up R	CATTATCCATTAAAAATCAAACGGATCCTAatatcctgtacctgctactgctattttc
	ugd Dn F	CAAAAGCATAAGGAAAGGGGCCCtatactagggatatttttaagagagattaaatc
	ugd Dn R	Cggaagacttgaaacaccacta
Δgla	gla Up F	Ggtggagttggtttaggtcag
	gla Up R	CATTATCCATTAAAAATCAAACGGATCCTAtcctgtaattagaattgtttttcctctc
	gla Dn F	CAAAAGCATAAGGAAAGGGGCCCgagtggtataaagactactataatgtataacg
	gla Dn R	Tgcctacataagatccagcaag

### Immunofluorescence microscopy and dot blots

Cultures were grown to an OD_600_ of 0.2 to 0.4, normalized to an OD_600_ of 0.2, and heat-inactivated at 65°C for 45 min. The cells were harvested by centrifugation at 20,000 ×*g* for 2 min at room temperature and washed once with 1 mL of 1× PBS. The cells were then resuspended in 100 µL of cross-adsorbed anti-CPS1 antisera at a dilution of 1:600, and the mixture was incubated on ice for 5 min. The cells were washed twice in 1× PBS and centrifuged at 20,000 × *g* for 2 min before resuspending in 100 µL of 1× PBS. Anti-rabbit Alexa Fluor 488 antibodies (Thermo Fisher Scientific, A11034) were added at a dilution of 1:100 on ice for 5 min. The labeled cells were washed once with 1× PBS and resuspended in 3 µL of SlowFade mounting medium (Invitrogen, S36936) and visualized by epifluorescence microscopy.

Strains D39::cps1, D39::cps1Δ*wchB*, D39::cps1Δ*wchC*, D39::cps1Δ*ugd*, and D39::cps1Δ*gla* were grown in BHI broth at 37°C in 5% CO_2_ until they reached the early to mid-exponential phases (OD_600_ between 0.2 and 0.4). 10 µL of each of the bacterial suspensions was spotted in NCM membrane and allowed to air-dry. D39::CPS1 and mutants were stained with anti-serotype1 sera (Statens, Denmark), followed by Alexa Fluor anti-rabbit secondary antibodies. D39::CPS1 and mutants were stained with anti-serotype2 antisera, followed by Alexa Fluor anti-rabbit secondary antibodies.

Anti-serotype 2 has wide reactivity and recognizes surface-bound proteins, and therefore all probed strains and corresponding mutants reacted with it including the negative acapsular R6 strain.

### Capsule thickness semi-quantification by FITC-dextran

**Figure 5:**
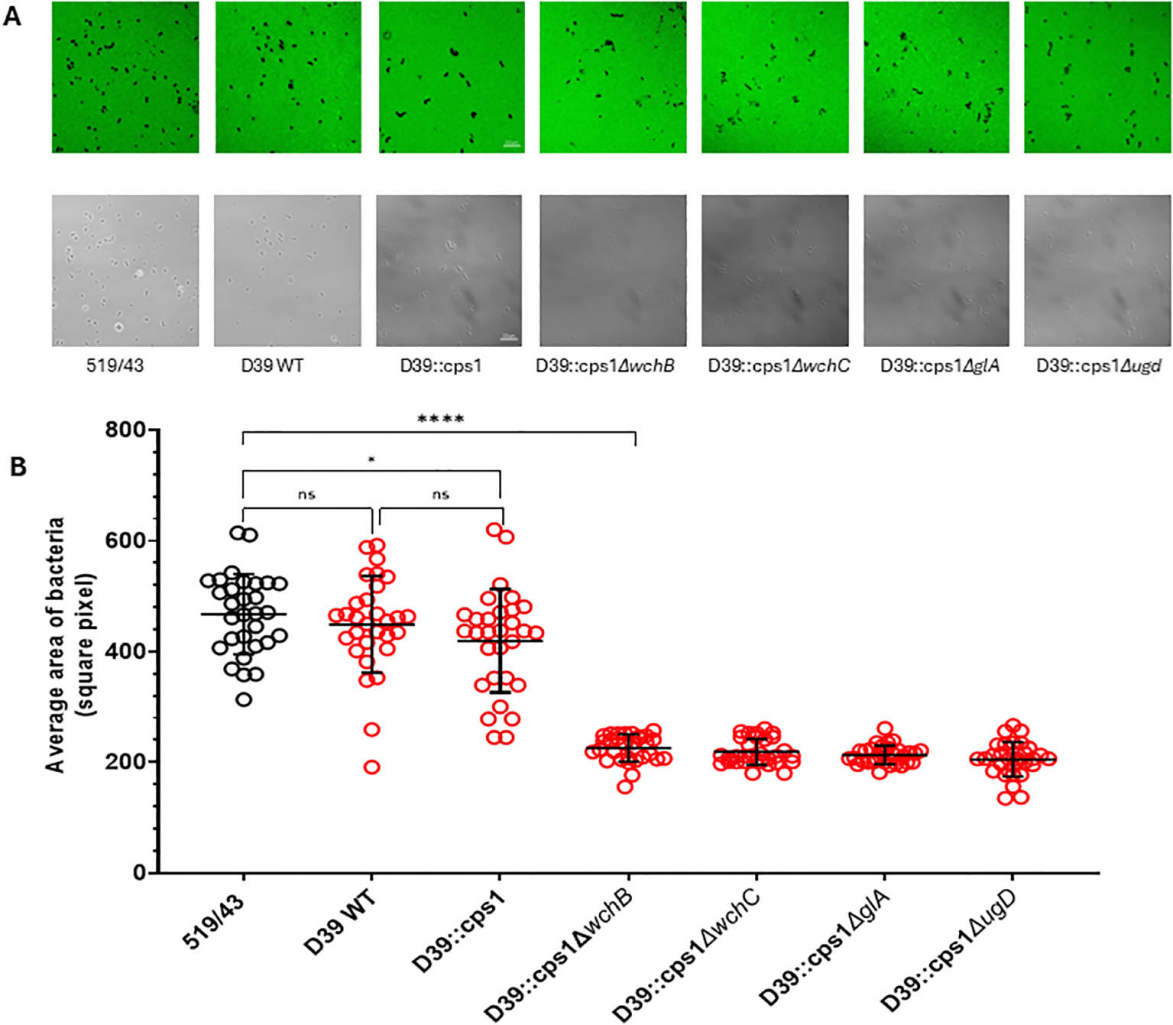
**(A)** Fluorescence microscopy of the FITC–dextran exclusion assay showing representative fields for S. pneumoniae 519/43 (serotype 1), D39WT (parental), and D39 carrying a serotype 1 capsule switch D39::cps1 and indicated capsule gene mutants. Cells appear as dark silhouettes within the fluorescent dextran background; stronger capsule/exclusion phenotypes present as larger, sharper dextran-free halos surrounding cells. **(B)** Quantification of dextran-exclusion–defined cell size across strains, demonstrating capsule-dependent increases in apparent cell size for serotype 1, D39 WT, and D39::cps1 and reductions for capsule mutants. Points represent individual cells (pooled from three independent experiments). Statistical tests and n=30; significance thresholds are denoted on the plot.

Capsule thickness was quantified by assessing the exclusion zone of FITC-dextran, following the methods described by Gates et al. (2004) (Gates et al., 2004) and Nasher et al. (2018) (Nasher et al., 2018), with minor modifications. Bacterial cultures were grown overnight in BHI supplemented with fetal calf serum (BHI+FCS) to an OD_600_ = 0.5 and then diluted 1:20 into fresh BHI and cultured further until reaching an OD_600_ = 0.3. Cells were harvested by centrifugation at 3,000 ×g for 5 min and resuspended in 500 μL of PBS. A 10-μL aliquot of the bacterial suspension was mixed with 2 μL of 2,000-kDa FITC-dextran (10 mg/mL in water; Sigma-Aldrich) and applied to a microscope slide, and a coverslip was applied firmly. Fluorescence imaging was performed on a Zeiss LSM880 confocal microscope using a 100× objective lens, with excitation at 488 nm (green channel). Images were converted to grayscale and analyzed using UTHSCSA ImageTool for Windows v3.0 (University of Texas Health Science Center, San Antonio, TX, USA). For each fluorescent image, a corresponding brightfield image was acquired to facilitate bacterial counting. Between 23 and 81 bacteria were present per image, and 10 images per group were analyzed in each of three independent experiments, resulting in 30 images per group. Each data point in **Figure 5** corresponds to one analyzed image. In this assay average area in square pixels indicates thickness of capsule.

### Scanning electron microscopy of D39 WT, D39::cps1, D39::cps1Δ*wchB*, and D39::cps1Δ*wchC*

*S. pneumoniae* cultures were grown to mid-log phase in Todd Hewitt + 0.5% yeast extract broth and prepared for SEM as previously described. Briefly, cultures were spun slowly to achieve a fluffy pellet and resuspended in fixative on ice for 1 h (0.1 M NaCAC buffer (0.1 M sodium cacodylate, 0.09 M sucrose, 0.01 M MgCl2, 0.01 M CaCl2, pH 6.9), 2% formaldehyde (Merck), 2.5% glutaraldehyde (TAAB), 0.075% ruthenium red (TAAB), 0.075 M L-lysine acetate). Samples were spun slowly to achieve a fluffy pellet and washed twice with wash buffer (0.1 M NaCAC buffer/0.075% Ruthenium Red). Samples were resuspended in fixative (without L-lysine acetate) and incubated on ice for 3 h followed by three washes. Samples were spun and osmicated in 1% OsO4/0.075% RR in 0.1 M NaCAC for 1 h at room temperature followed by 2× washes with wash buffer. Samples were dehydrated in ethanol (10%, 30%, 50%, 70%, 90%, 100%), with incubation for 30 min on ice for each step followed by infiltration with LR white hard resin (1:1 with ethanol for 2 h, 1:2 overnight on ice, pure LRW for 8 h on ice, pure LRW overnight at 4°C, pure LRW for 3 h at RT). Bacterial suspensions were embedded out in LRW using gelatine capsules. The resin was set under exclusion of air in an embedding oven at 55°C for 24 h. Sections were cut using a Leica Ultracut microtome and put on Melinex plastic coverslips. Melinex coverslips were mounted on aluminum SEM stubs using a carbon sticky and sputter-coater with 30-nm carbon to ensure conductivity. The coverslips were imaged in a FEI/Thermo Fisher Verios 460 SEM run at 4 keV accelerating voltage and 0.2-nA probe current using the concentric backscatter detector in immersion mode, with pixel resolution 1,536 × 1,024, 3-µs dwell time, and three- or four-line integrations. Stitched image maps were acquired using FEI/Thermo Fisher MAPS software. Capsule and cell areas were measured using ImageJ (v 1.54g).

### Complement deposition and antibody binding 519/43WT, D39WT and D39::cps1, and capsule isogenic mutants

Complement deposition and antibody binding to different strains of *S. pneumoniae* were assessed by flow cytometry, as previously described by Ramos-Sevillano et al. (Domenech et al., 2013), with minor modifications. Briefly, *S. pneumoniae* strains were grown to mid-logarithmic phase (OD_600_ ≈ 0.3), washed twice in phosphate-buffered saline (PBS), and adjusted to ~5 × 10^5^ CFU per sample. For IgG binding assays, bacteria were incubated for 30 min at 37°C with 10% or 2.5% intravenous immunoglobulin (IVIG; pooled human IgG from healthy donors, commercial preparation) in PBS. For C3b deposition, bacteria were instead incubated with 10% or 2.5% pooled normal human serum (NHS) as a complement source under the same conditions. After two washes with 1× PBST (0.1% Tween-20), strains were labeled with 50 µL of 1:200 APC-labeled anti-human IgG for IgG binding (BioLegend) or 50 µL of 1:300 FITC-polyclonal goat anti-human C3b antibody (ICN) in the dark for 30 min. Bacterial suspension was washed 2× and fixed in 50 µL 4% PFA in PBS. The suspension volume was adjusted up to 250 µL with 1× PBS prior before flow cytometry using a BD FACSVerse instrument. Data were analyzed using FlowJo software, and results are expressed as geometric mean fluorescence intensity (MFI). At least 10,000 events were recorded per sample.

### Zebrafish infection D39WT, D39::cps1, and D39::cps1*wchB*

The AB wild-type zebrafish (*Danio rerio*) larvae were infected and maintained for up to 5 days post fertilization (i.e., non-protected stage under the Animals Scientific Procedures Act 1986, ASPA). During the experiments, zebrafish were maintained in E3 medium and kept at 28 °C. At 2 dpf, zebrafish were anaesthetized with buffered 0.02% tricaine. 0.05% phenol red was added to the bacterial suspensions before injections. Zebrafish larvae were injected with 600 CFU *S. pneumoniae* serotype 2 carrying and expressing the serotype 1 capsule loci D39::CPS1, D39::CPS1Δ*wchB and* D39 WT in the hindbrain. After injected embryos were kept in 24-well plates at 28°C, the embryos were checked every 24 h and any dead embryos were counted at each time point to obtain accurate survival rates. Lack of heart beat was counted as dead. Three embryos were collected at time 0 to determine the injection dose. Any remaining larvae were humanely sacrificed at 5 dpf as the experimental end was reached. A survival curve was plotted, and survival rates were analyzed using the Mantel–Cox log rank test.

## Results

### Mutagenesis of the *cps* loci in *S. pneumoniae* serotype 1

Mutagenesis of the *cps* loci in 519/43 strain proved extremely challenging. Mutation of genes *wzg, wchB, wchC, wzy, gla*, and *ugd* was unsuccessful despite more than 10 attempts per target. However, genes contained within the *cps* loci that do not directly code for the assembly or generation of the monosaccharides present in the serotype 1 capsule were readily disrupted. Genes targeted include *rlmA*, *rlmB*, *rlmC*, and cpsO1. Mutation of gene *swt00335* was also successful (**Figure 1**).

### Confirmation of loss of capsule by the D39::cps1 mutants via immunofluorescence microscopy

Strains D39::cps1, D39::cps1Δ*wchB*, D39::cps1Δ*wchC*, D39::cps1Δ*ugd*, and D39::cps1Δ*gla* were probed to confirm the presence of serotype 1 capsule on D39::cps1 as well as the loss of capsule in the capsule mutants generated in this background (**Figure 4**).

As expected, D39::cps1 reacted with the anti-serotype 1 antisera. Mutants in genes *wchB* (responsible for the addition of the galacturonic acid to AATGal), *ugd* (synthesis of glucuronic acid), and *gla* (conversion of glucuronic acid to galacturonic acid) yielded mutant strains that did not react with the anti-serotype 1 antisera. The mutant in *wchC* responsible for the acetylation of AATGal presented with an intermediate phenotype, where 54.9% of the bacteria reacted with the anti-sera (n=253). The anti-serotype 2 antibody was also used to probe bacteria; however, anti-serotype 2 antibody exhibits reactivities with surface-exposed proteins, and therefore, all strains reacted with it. R6, a known unencapsulated serotype 2 strain, was added as the negative control.

### Capsule thickness semi-quantification by FITC-dextran exclusion

Capsule thickness was quantified using FITC-dextran exclusion (**Figure 5**). The data demonstrated that there is no significant difference between the capsule thickness of D39WT when compared with D39::cps1, the capsule switch strain. However, there is a significant difference between 519/43 WT strain when compared with D39::cps1 (p<0.05). 519/43 WT presents a thicker capsule (466.77 square pixel) than D39::cps1(407.06) after growing in BHI. D39 parental strain had a capsule thickness of around 450.1 square pixel. Mutants D39::cps1Δ*gla* presented the least thickness at just 200.26 square pixels, whereas D39::cps1Δ*ugd*, D39::cps1Δ*wchC*, and D39::cps1Δ*wchB* capsule thicknesses were respectively 206.47, 215.70, and 235.26. All mutants presented significantly smaller areas p<0.0005 when compared with all wild types included in the experiment, suggesting that the mutants had either lost their capsule or had just one unit of UndPP decorated with AATGAl. Of note is the fact that the CPS thickness measured by the FITC-dextran exclusion assay is consistent with the dot blot results, indicating that the amount of CPS is comparable between the D39::cps1 mutant and 519/43.

### Scanning electron microscopy for determination of presence/absence of cell capsule on D39::cps1, D39 WT, and mutants D39::cps1Δ*wchB* and D39::cps1Δ*wchC*

Electron microscopy was performed on the capsule-switch strain D39::cps1, the parental D39 wild-type (WT) strain, and the isogenic mutants D39::cps1Δ*wchB* and D39::cps1Δ*wchC*. The analysis revealed that D39::cps1 exhibited a statistically significant thicker capsule compared with the D39 WT parental strain. Consistent with the areas quantified using FITC–dextran exclusion, both capsule mutants displayed significantly smaller capsule areas than either the parental D39 WT or the capsule-switch strain (p < 0.0001).

**Figure 6:**
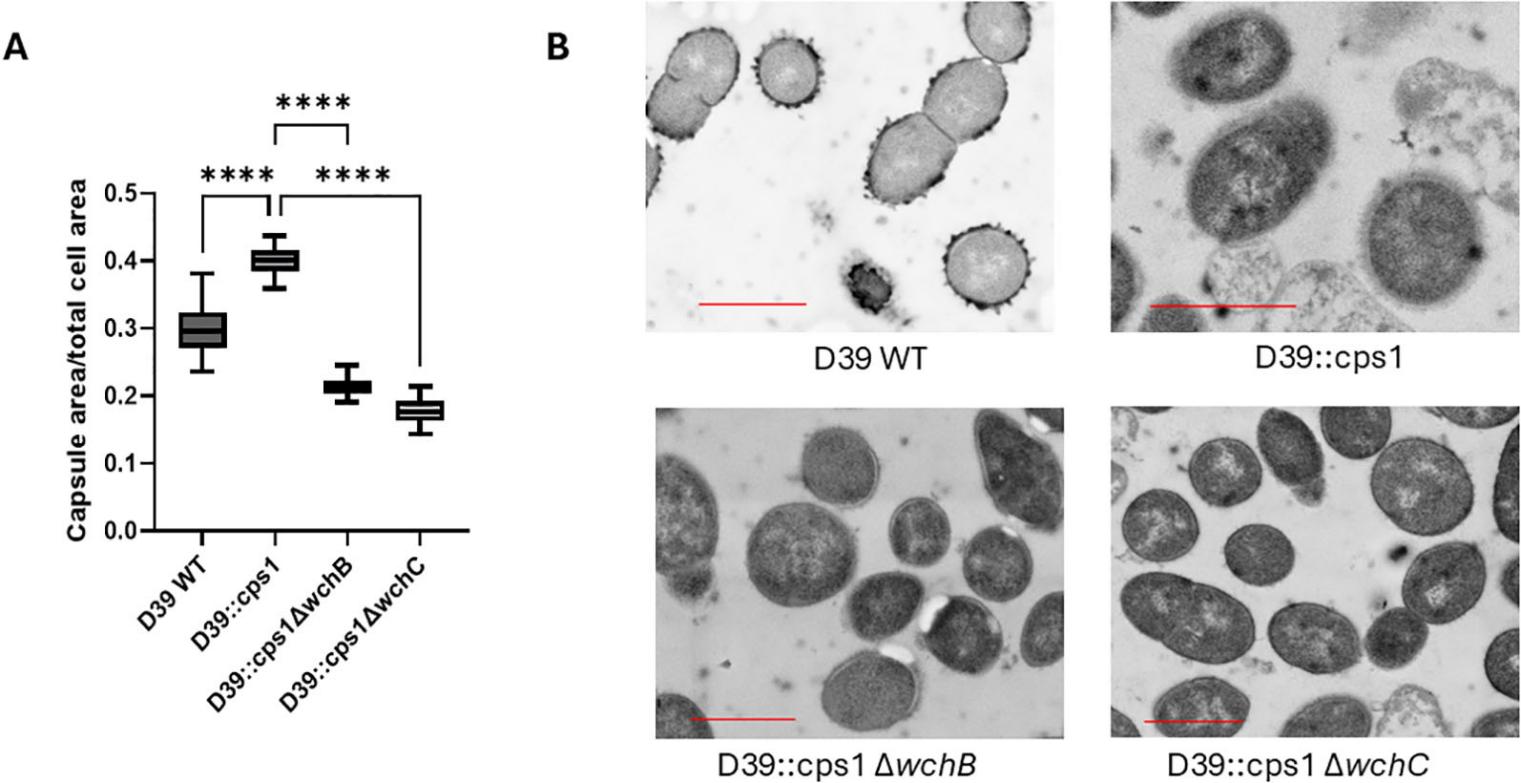
**(A)** Ratio of capsule area vs. total cell area for S. pneumoniae D39, D39::CPS1, D39ΔwchB, and D39ΔwchC measured by SEM. n=30 per strain. One-way ANOVA, ****P<0.0001. **(B)** Typical SEM images of S. pneumoniae D39, D39::CPS1, D39ΔwchB, and D39ΔwchC. Scale bar represents 1 µm.

Notably, a clear morphological difference was observed between the homologous serotype 2 capsule, characterized by a “spiky” surface phenotype (**Figure 6B**, top left), and the heterologous serotype 1 capsule expressed by D39::cps1, which appeared denser and more uniform in thickness (**Figure 6B**, top right). The characteristic “fuzzy” appearance associated with capsule presence was visibly absent in both mutant strains, consistent with the expected absence of capsule in D39::cps1Δ*wchB* and the presence of a reduced, non-polymerized capsule in D39::cps1Δ*wchC* (**Figure 6B**, bottom panels).

### C3b deposition and IVIG

To assess the impact of capsule expression and specific capsule-associated gene deletions on IgG binding and complement deposition, we evaluated a panel of *S. pneumoniae* strains using IVIG and C3b deposition assays. The strains included ST1 (519/43), D39_S1, a D39 capsule switch expressing the ST1 capsule (D39::cps1), and four isogenic D39 mutants carrying single-gene disruptions within the capsule operon, resulting in unencapsulated phenotypes.

**Figure 7:**
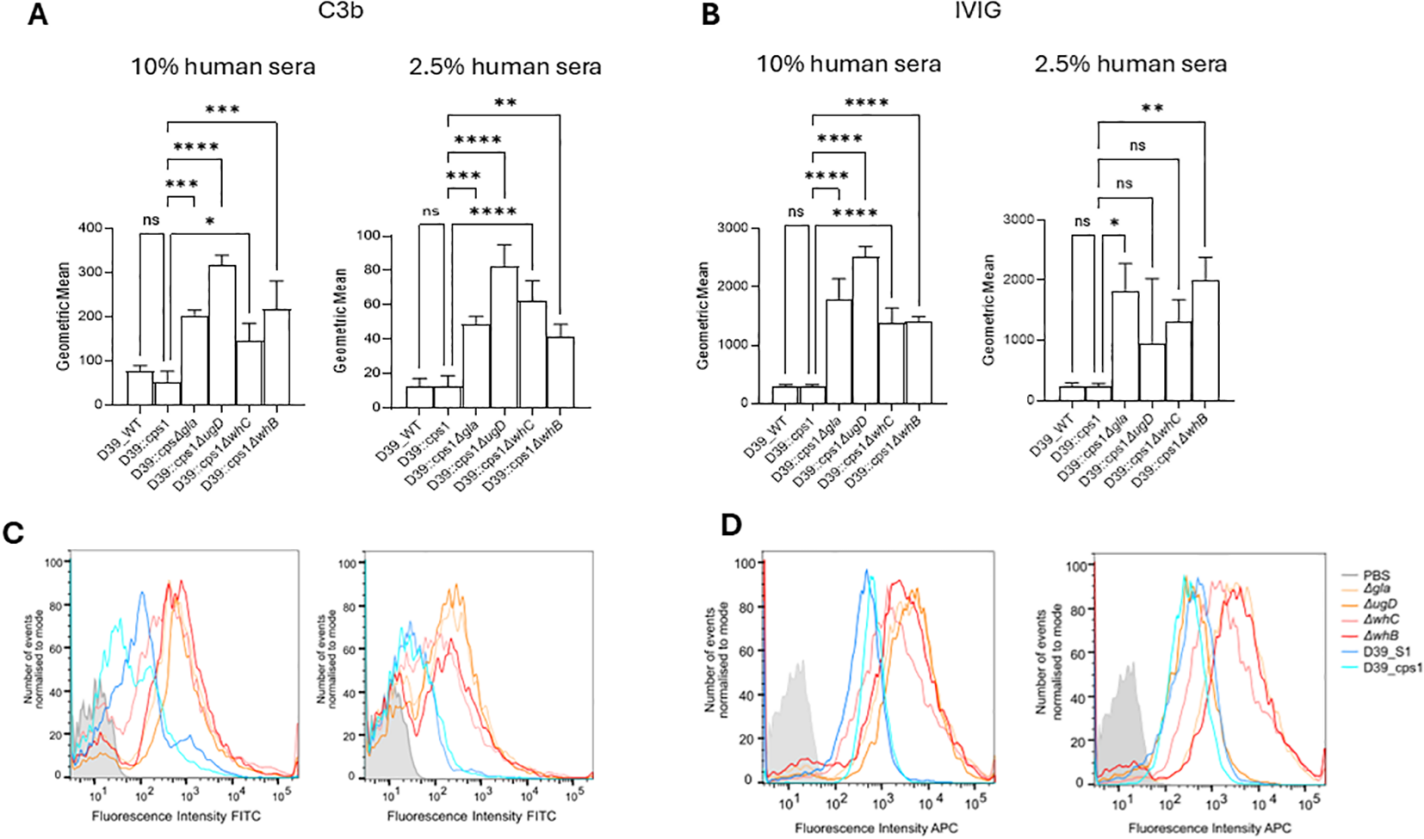
C3b and human IVIG deposition on Streptococcus pneumoniae D39 and capsule mutant strains. **(A)** Geometric mean fluorescence intensity (gMFI) of C3b deposited on the surface of wild-type D39 and capsule mutant strains following incubation with 10% or 2.5% human serum, as measured by flow cytometry. **(B)** Geometric mean fluorescence intensity (gMFI) of human IVIG deposited on the surface of wild-type D39 and capsule mutant strains following incubation with 10% or 2.5% human serum, as measured by flow cytometry. **(C)** Representative flow cytometry histograms showing C3b and **(D)** IVIG **(D)** deposition. Histograms display the number of events normalized to mode versus fluorescence intensity for FITC (C3b) or APC (IVIG). Error bars **(A, B)** represent standard deviation, and asterisks represent statistical significance compared with the wild-type strain (two-sided Kruskal–Wallis test with Dunn’s correction for multiple comparisons) *p < 0.05; **p < 0.01; ***p < 0.001; ****p < 0.0001).

Flow cytometry revealed that both IVIG binding and C3b deposition were significantly increased in the unencapsulated mutants compared with encapsulated D39WT and D39::cps1 strains (**Figure 7**). These findings indicate that the loss or severe reduction of capsule, even due to single-gene mutations within the operon, substantially enhances recognition by pooled human IgG and facilitates complement component deposition. In contrast, encapsulated strains—including D39 expressing the heterologous ST1 capsule—showed reduced binding of both IVIG and C3b, consistent with the known protective role of the capsule in masking underlying antigens and inhibiting complement activation.

### Zebrafish infection D39WT, D39::cps1, and D39::cpsΔ*wchB*

**Figure 8:**
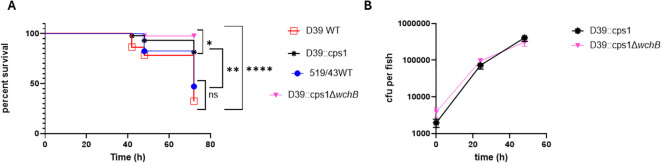
**(A)** Survival of zebrafish larvae when infected with S. pneumoniae strains 519/43 WT, D39WT, D39::cps1, and D39::cps1ΔwchB. Zebrafish larvae injected at 2 dpf and followed until 5 dpf. n ≥40 for the mutants for confirmation of decreased virulence, over three independent experiments. **(B)** Bacterial loads were calculated for D39::cps1 and D39::cps1ΔwchB to confirm that diminished virulence was not due to a growth defect. *p < 0.05; **p < 0.01; ****p < 0.0001.

2dpf zebrafish larvae were injected with ≈1,000 CFU of either 519/43 WT, D39WT, D39::cps1, and D39::cpsΔ*wchB*, directly in the hindbrain ventricle. Experiments were terminated at 5 dpf (i.e., non-protected stage under the Animals Scientific Procedures Act 1986, ASPA). The data demonstrated that despite the expression of the heterologous serotype 1 capsule, virulence of D39::cps1 was significantly different of that of 519/43WT (p<0.01) or D39WT (p<0.0001, **Figure 8**). Zebrafish larvae infected with D39::cps1 had a survival of 81.4% (n=42), which was significantly different from the survival rate of zebrafish larvae infected with D39::cps1Δ*wchB* (97.6% n=41) (**Table 6**). Bacterial loads for zebrafish infected with D39::cps1 and D39::cps1Δ*wchB* calculated by sacrificing zebrafish at 0, 24, and 48 h. Bacterial loads in zebrafish infected with D39::cps1 were 1.97 × 10^3^ ± 4.93 × 10^2^, 7.27 × 10^4^ ± 1.53 × 10^4^, and 4.1 × 10^5^± 8.94 × 10^4^ cfu/fish at 0, 24, and 48 h, respectively, and bacterial loads in zebrafish infected with D39::cps1Δ*wchB* were 3.87 × 10^3^ ± 1.10 × 10^3^, 9.77 × 10^4^ ± 4.04 × 10^3^, and 3.10 × 10^5^ ± 7.00 × 10^4^ cfu/fish at 0, 24, and 48 h, respectively.

**Table 6:** Survival of Zebrafish larvae when infected with 519/43 WT, D39WT, D39::cps1 and D39::Cps1Δ*wchB*.

Strains	Survival %			
	24h	42h	48h	72h
519/43WT	100	100	82.4	47
D39WT	100	86.5	78.4	32.4
D39::cps1	100	97.7	93	81.4
D39::cps1Δ*wchB*	100	100	97.6	97.6

Survival of zebrafish larvae following infection with 519/43 WT, D39 WT, D39::cps1, and D39::Cps1Δ*wchB*. Survival was defined as the proportion of larvae alive at each observation timepoint relative to the initial number per group. Larvae were considered alive if a heartbeat was observed.

## Discussion

*Streptococcus pneumoniae* serotype 1 strains possess a capsule that is structurally and genetically distinct from those of most other pneumococcal serotypes (Bentley et al., 2006) (Tettelin et al., 2001). The serotype 1 capsule is zwitterionic (Cobb and Kasper, 2005) and composed of only two different sugars, AATGal and galacturonic acid, distinguishing it from the more complex polysaccharides found in other serotypes (Bentley et al., 2006). Moreover, this capsule is anchored to undecaprenyl pyrophosphate (UndPP) via AATGal, with the corresponding biosynthetic genes located outside the conventional capsule locus typically flanked by *dexB* and *aliA* (Aanensen et al., 2007). Notably, the enzymes responsible for the synthesis and transfer of AATGal are shared with the teichoic and lipoteichoic acid biosynthetic pathways, both of which play essential roles in cell wall architecture and integrity (Behr et al., 1992; Fischer et al., 1993). Given that tools to genetically manipulate *S. pneumoniae* serotype 1 strains have been recently developed (Terra et al., 2020b, 2020a) and that multiple mutants in distinct genes can be generated (e.g., *ply*, *bgaA* etc.), and considering that the capsule is a major virulence determinant of *S. pneumoniae* with unique biochemical and genetic features, we undertook a comprehensive analysis of the corresponding genomic region by attempting to generate isogenic mutants. This approach aimed to clarify the genetic basis and potential functional implications of the distinctive serotype 1 capsule features in pneumococcal pathogenesis.

Mutagenesis of genes within the *dexB–aliA* region, responsible for serotype 1 capsule assembly, were unsuccessful in the parent strain 519/43 despite extensive efforts. This finding suggests that several genes within this locus may be essential for bacterial viability or that disruption of capsule biosynthesis in serotype 1 has lethal consequences. The mutagenesis strategy followed a logical sequence, beginning with *wzg*, a regulatory gene whose deletion in other serotypes results in a significant expression reduction of the capsule polysaccharide (Morona et al., 2000, 2003, 2004). All attempts to disrupt *wzg* in serotype 1 failed to yield viable transformants, indicating that *wzg* may play an essential or uniquely adapted role in this serotype.

Similarly, attempts to inactivate *wchB*, which encodes the enzyme responsible for adding galacturonic acid to the AATGal sugar, produced no viable mutant colonies in either strain 519/43 or BVJ1JL. This was unexpected, as loss of *wchB* was hypothesized to generate a truncated AATGal-only capsule. The inability to disrupt *wchB* suggests that this enzymatic step may be critical for maintaining capsule integrity and/or for the stability of the cell envelope in serotype 1.

Further targeting of *wchC*, encoding a capsule acetyltransferase, also failed to yield mutants, even when in-frame mutagenesis was employed to preserve the polycistronic structure. Given that capsule acetylation is absent in most serotype 1 isolates (Bentley et al., 2006), the lack of viable *wchC* mutants was unexpected and may indicate strain-specific dependence on capsule modifications. Likewise, attempts to disrupt *wchD* were unsuccessful, again suggesting that interference with early or intermediate steps of capsule assembly leads to non-viable bacterial cells.

Broad-scale deletion of the *aliA–dexB* region and targeted disruption of *ugd_2*—the terminal enzyme in the serotype 1 capsule pathway—also failed to produce viable transformants.

Collectively, these results suggest that mutagenesis of individual genes, or of the capsule locus, is not possible. One possible explanation is the unavailability of undecaprenyl. If the capsule repeat unit is not assembled, it is unlikely to be polymerized even if it is flipped across the membrane. Consequently, UndPP-AATGal may remain bound, preventing efficient recycling of undecaprenyl. If this occurs, UndPP would not be available for the synthesis of lipoteichoic acids (LTA) or wall teichoic acids (WTA), which could have fatal consequences for the cell.

Alternatively, the UndPP-AATGal complex may still be assembled but not shared between the capsule biosynthesis pathway and the WTA/LTA pathways. This could lead to dysregulation and excessive production of WTA and LTA, which may also be detrimental to the bacterial cell, since excessive WTA induces autolysis via lytA (Flores-Kim et al., 2022). Together, these possibilities suggest that the “WTA capsule,” in which the main precursor is shared between pathways, represents an intrinsic vulnerability and that in serotype 1, capsule assembly and cell viability are more interconnected than in other pneumococcal serotypes.

To address this limitation, we adopted two alternative strategies, one where we expressed the serotype 1 capsule loci in an *E. coli* strain for systematic mutagenesis, and a second approach where we used a previously generated capsule-switch mutant D39::cps1 (Chun et al., 2023), which enables the expression of the serotype 1 capsule within the well-characterized D39 strain background. Given that mutagenesis of pneumococcal *wchB* expressed in the *E. coli* background created an acapsular mutant essentially creating an acapsular *E. coli* strain (data not shown), we have abandoned this strategy to focus on the capsular switch mutant D39::cps1. This system provided a robust genetic framework in which corresponding acapsular mutants could be generated and compared under controlled conditions. To validate this model, we performed a series of phenotypic assays to assess whether the D39::cps1 strain recapitulates the characteristics of the wild-type 519/43 isolate. Quantitative analysis of capsule thickness revealed that the 519/43 WT serotype 1 strain possesses a significantly thicker capsule than D39::cps1, whereas the capsule of D39::cps1 did not differ significantly from that of the D39 WT parental strain. This suggests that, although the serotype 1 polysaccharide can be successfully expressed in the D39 background, its synthesis or polymerization is influenced by strain-specific factors inherent to the host strain (Hathaway et al., 2016) (Hathaway et al., 2012).

Moreover, the acapsular mutants exhibited a markedly reduced area of exclusion, consistent with either the complete loss of capsule or defective polymerization, confirming the functional disruption of capsule biosynthesis pathways (**Figure 5**). To further examine these structural differences, SEM was used to compare the morphology of the serotype 1 capsule produced by the wild-type strain with that expressed by D39::cps1. The observed ultrastructural differences highlight how the capsule’s density may be modulated by the genetic background (Hathaway et al., 2012).

We then investigated if expressing a heterologous capsule would result in higher or lower C3b deposition when compared with the capsule wild-type strain 519/43WT and the background strain D39WT. We observed that irrespective of the background in which the serotype 1 capsule was expressed, its presence alone had the expected phenotype of decreased c3b deposition (Melin et al., 2010b). Similarly, any of the mutants had a significantly higher c3b deposition when compared with the WTs. The same phenotype was observed when investigating antibody binding; the presence of capsule decreased the ability of antibody to bind to the bacteria in line with what is expected.

We then used an established zebrafish infection model to assess if D39 heterologously biosynthesizing serotype 1 capsule would show similar virulence to either of the parental strains 519/43 or D39WT. While the zebrafish model is highly informative for studying bacteremia and meningitis, it does not fully recapitulate all aspects of human pneumococcal disease. In particular, differences in host physiology, immune responses, and route of infection should be considered when interpreting these findings. Despite these limitations, the D39::cps1 did not exhibit virulence similar to neither of the parental strains and instead showed reduced virulence. D39::cps1 was still more virulent than the acapsular isogenic mutant (p<0.05). Similar phenotypes were previously reported. For example, Kadioglu et al. (2002) reported that a D39 strain expressing the capsule of a serotype 3 strain (A66) did not exhibit virulence similar to neither of the parental strains (D39~45h, A66~69h), with all mice surviving for 7 days (Kadioglu et al., 2002). Additionally, Sanchez et al. reported a significant decrease in virulence when TIGR4 serotype 4 strains expressing capsule types of 6A,7F, and 23F were used in an intratracheal mouse model, with only a restored type 4 capsule behaving as the original strain (Sanchez et al., 2011). Similarly, Hathaway et al. demonstrated that a serotype 6B strain or its back transformant caused more severe meningitis than when a 6B strain background was expressing a 7F capsule (Hathaway et al., 2016). How the background influences the virulence of the strain is not yet fully understood; however, it could be due to different factors. For example is the capsule thickness. In this study, we have reported a significantly thinner capsule for D39::cps1 when compared with 519/43 using FITC dextran exclusion and a significantly reduced virulence. The extent to which strain background or the physical properties of the capsule contribute to virulence remains to be conclusively determined. However, evidence from our work and that of others indicates that expression of a capsule on a heterologous strain background does not fully recapitulate the virulence of the original capsule donor strain. This occurs despite the retention of several capsule-associated phenotypes, including increased resistance to complement deposition and reduced antibody-binding capacity.

## Conclusion

Mutagenesis of genes required for synthesis and assembly of the serotype 1 capsule was unsuccessful in both serotype 1 strains tested, suggesting that this capsule may be indispensable for bacterial survival. In contrast, when the serotype 1 capsule locus was introduced into the serotype 2 D39 background, mutation of the same genes was successful and yielded acapsular isogenic mutants. Although D39 was able to express the serotype 1 capsule and its presence enhanced resistance to complement deposition and antibody binding, it did not increase virulence. Instead, virulence of the D39::cps1 strain was significantly reduced compared with both D39 WT and the 519/43 WT strain. Together, these findings indicate that the role of the serotype 1 capsule in pneumococcal survival and pathogenesis is more complex than anticipated. Critically, this highlights the need for improved molecular tools—particularly approaches that enable gene knockdown rather than complete knockout—which will be essential to fully elucidate the function of this capsule while preserving bacterial viability. Furthermore, advancing our understanding will require the use of colonization or intranasal infection models to assess the ability of D39::cps1 to cross the nasal mucosa and contribute to the development of pneumonia and/or bacteremia.

## Data Availability

The raw data supporting the conclusions of this article will be made available by the authors, without undue reservation.
